# The Effect of Nonsteroidal Anti-inflammatory Drugs on Union Rates Following Joint Arthrodesis: A Meta-Analysis

**DOI:** 10.7759/cureus.56312

**Published:** 2024-03-17

**Authors:** Emerson T Rowe, Julian Takagi-Stewart, Sina Ramtin, Margaret Pennington, Asif M Ilyas

**Affiliations:** 1 Orthopedic Surgery, Drexel University College of Medicine, Philadelphia, USA; 2 Hand Department, Rothman Orthopaedic Institute, Philadelphia, USA; 3 Division of Hand Surgery, Rothman Orthopaedic Institute at Thomas Jefferson University, Philadelphia, USA

**Keywords:** non-steroidal anti-inflammatory, opioids, pain management, nonunion, malunion, arthrodesis

## Abstract

Nonsteroidal anti-inflammatory drugs (NSAIDs) are among the most widely used and prescribed medications because of their important role in reducing inflammation and pain, in addition to their non-addictive properties and safety profiles. However, some studies have documented an association between NSAIDs and delayed union or nonunion of joint arthrodesis procedures due to a potential inhibition of the bone’s inflammatory healing response. As a result, some orthopedic surgeons hesitate to prescribe NSAIDs after an arthrodesis procedure. The purpose of this meta-analysis is to review all relevant literature regarding the effect of NSAIDs on union rates after arthrodesis and determine if NSAID therapy increases the risk of non-union in the setting of arthrodesis procedures. The study hypothesis was that NSAIDs would not have a significant effect on the risk of nonunion after arthrodesis.

A thorough systematic review of Medline, Embase, the Cochrane Database of Systematic Reviews, and the Web of Science identified 3,050 articles to be screened. The variables of interest encompassed demographic factors, procedural details, type and administration of NSAIDs, the number of patients exposed to NSAIDs with and without successful union (case group), as well as the number of patients who did not receive NSAIDs with and without successful union (control group). All the data were analyzed using a maximum likelihood random-effects model. The number of non-union events versus routine healing from each study was used to calculate the odds ratio (OR) of successful healing after arthrodesis procedures with versus without NSAID therapy.

Thirteen articles met the inclusion criteria for the meta-analysis. NSAID exposure showed an increased risk of nonunion, delayed union, or both following arthrodesis procedures; however, this did not meet statistical significance (OR, 1.48; confidence interval [CI], 0.96 to 2.30). A sub-analysis of pediatric and adult studies showed a significant increase in non-union risk in adults (OR, 1.717; CI, 1.012 to 2.914) when removing the pediatric cohort (p = 0.045).

This meta-analysis provides evidence that NSAIDs can increase the risk of nonunion, delayed union, or both following arthrodesis procedures in adults. However, the study did not identify a risk of nonunion, delayed union, or both following arthrodesis procedures in the pediatric population.

## Introduction and background

Nonsteroidal anti-inflammatory drugs (NSAIDs) have gained popularity as a favorable option for managing pain and inflammation owing to their non-addictive properties and low-risk safety profiles [1]. This is particularly significant in light of the opioid epidemic and specifically with the increase in opioid-related deaths, with more than half a million people succumbing to prescription or illicit opioid overdoses since 1999 [2]. The surge in opioid-related death rates emphasizes the need for safer pain management protocols that reduce reliance on opioids and mitigate associated risks such as addiction, morbidity, and mortality [3].

As the development of multimodal pain management protocols has gained momentum, the use of NSAIDs has emerged as an appealing option due to their over-the-counter availability and relatively fewer side effects compared to other medications for pain control [4]. However, a relevant concern among orthopedic surgeons is whether the use of NSAIDs for postoperative pain may compromise bone healing, particularly following joint arthrodesis procedures, due to their potential inhibition of the bone's normal inflammatory healing response [5,6]. The inhibitory effect of NSAIDs on cyclooxygenase (COX) enzymes, which are responsible for producing prostaglandins that modulate bone formation and healing, has raised valid suspicions regarding their impact on the routine bone healing process [7]. Nevertheless, the existing literature on the effects of NSAIDs on various bone pathologies is inconclusive [8].

At the cellular level, Pountos et al. conducted a study on osteogenic and chondrogenic mesenchymal stem cell proliferation and differentiation in the presence of COX inhibition [9]. Their research indicated that seven COX-1 and COX-2 inhibitors did not inhibit proliferation or osteogenic differentiation at concentrations ranging from 10-3 to 100 µg/mL. While diclofenac and ketorolac showed some inhibitory effects on chondrogenic differentiation, COX-2 inhibitors parecoxib and meloxicam exhibited fewer inhibitory effects on this process. These findings suggest that selective COX-2 inhibitors could have a reduced inhibitory effect on endochondral bone formation [9]. Studies in animals treated with NSAIDs to observe successful bony arthrodesis have raised concerns, as they demonstrate an increased risk of impaired bone healing in the cohort who received NSAIDs [10]. However, results from clinical studies on patients undergoing spinal arthrodesis procedures with versus without post-operative NSAIDs showed that there was no clinical evidence to support the categorical discard of NSAIDs for pain relief in uncomplicated cases [11]. Moreover, a shift in thinking in recent years to questioning the true effect of NSAIDs on bone healing has prompted the question of whether studies conducted before 2005 had sufficient means to determine the true association of NSAIDs with the rate of non-union after arthrodesis [8].

The adverse effects and high-risk profile of opioid medication have fueled the search for enhanced multimodal pain management strategies, with a focus on NSAIDs as a potential alternative [12]. However, the potential inhibition of bone healing following arthrodesis procedures necessitates a thorough review of recent literature to establish a consensus on NSAID safety in the post-arthrodesis setting. The most current meta-analysis that reviews the effect of NSAIDs on bone healing after arthrodesis uses studies that compiled data until October 2016 [13]. This meta-analysis suggested an overall negative effect of NSAIDs on bone healing. However, this study noted limitations due to the lack of high-quality studies such as randomized controlled trials, the inclusion of studies involving pediatric patients, and considerable variability between joint arthrodesis, surgical techniques, and treatment variables between studies [13]. Additionally, the meta-analysis encompassed data from fractures, arthrodesis, and osteotomies combined, making it challenging to isolate the specific effect of NSAIDs on the risk of nonunion after an arthrodesis procedure. This is important to distinguish because the physiology of healing after fracture versus arthrodesis procedures differs, with arthrodesis typically entailing longer healing times, surgically induced inflammation, higher complication rates, and more chronic pathologies leading to surgery [14]. On the other hand, fractures have shorter healing times, encounter natural-onset inflammation if managed nonoperatively, have lower complication rates, and result from acute trauma [15].

To address these limitations and expand the current knowledge base, this study aims to conduct an up-to-date meta-analysis focused solely on arthrodesis procedures. The study hypothesis was that NSAIDs will not have a significant effect on the risk of nonunion after arthrodesis compared to the non-exposed control group and that NSAIDs may be utilized as a safe and effective alternative for pain management in the setting of arthrodesis procedures. Additionally, this study hopes to analyze pediatric and adult studies separately to validate any differences based on age.

## Review

Methodology

Literature Search

In June 2023, a comprehensive database search was performed for relevant literature published from January 1, 1990, to June 1, 2023. This search encompassed computerized databases, including MEDLINE (via Pubmed and Ovid), Embase, the Cochrane Database of Systematic Reviews, and the Web of Science. To ensure the retrieval of the most complete data on this topic, the lead authors identified key terms and Medical Subject Heading (MeSH) terms for this search (see appendix).

Inclusion and Exclusion Criteria

This meta-analysis included studies involving adult and/or pediatric patients who underwent arthrodesis and had been exposed to post-operative NSAIDs. The included studies needed a comparison to a control group that had not been exposed to NSAIDs. The primary outcome of interest was the occurrence of abnormal bone healing events, which encompassed nonunion, delayed union, and pseudoarthrosis. All studies included had a minimum follow-up duration of six months. All relevant randomized controlled trials, cohort studies, and case-control studies were included in this study.

Study Selection

**Figure 1:**
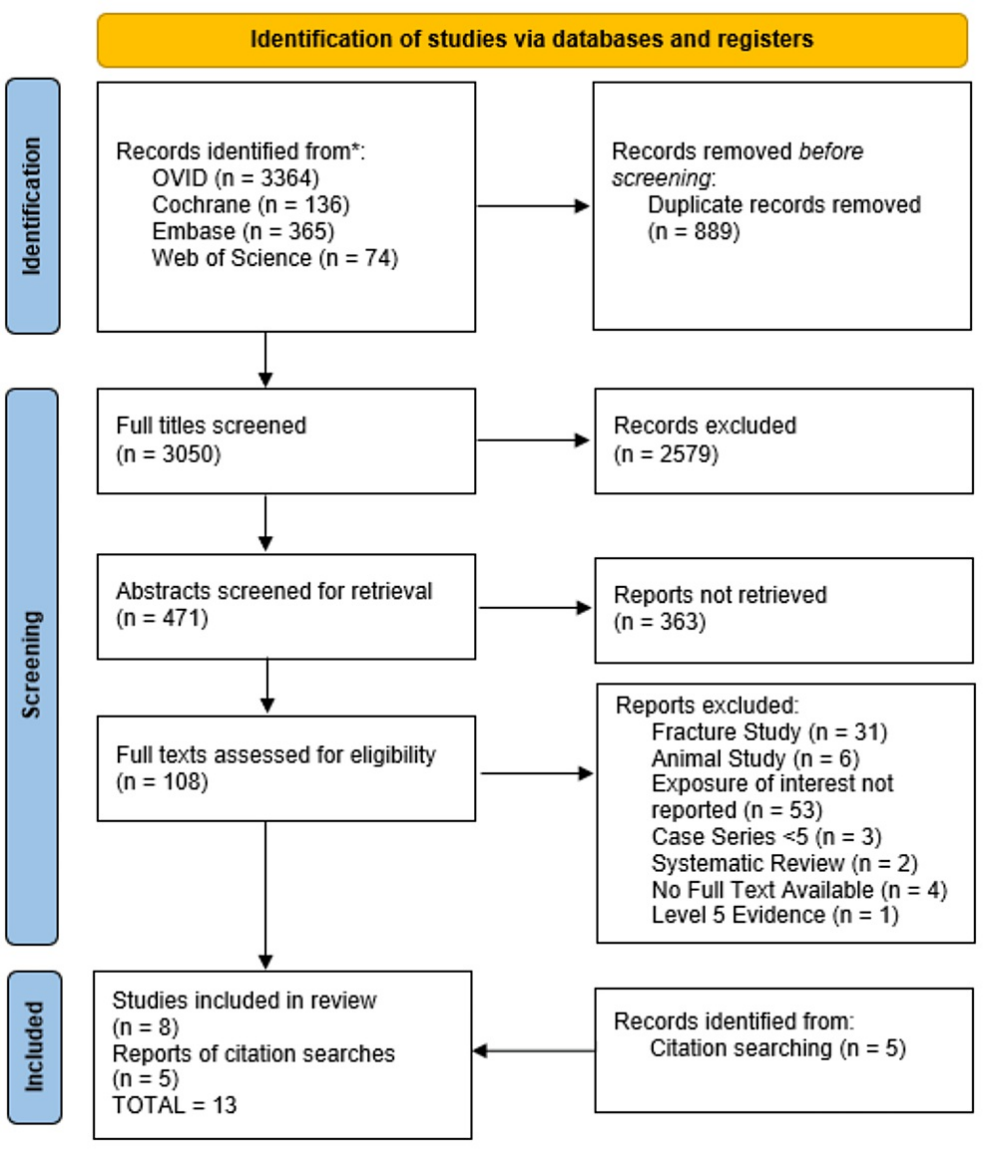
PRISMA flow chart of included studies PRISMA: Preferred Reporting Items for Systematic Reviews and Meta-Analyses

To ensure a rigorous selection process, each article underwent screening based on the predefined inclusion criteria (Figure 1). Independent evaluations by two of the authors (ER and JTS) were conducted sequentially, reviewing titles, abstracts, and full text to determine final inclusion. Articles were included when either one of the two reviewers deemed the article acceptable. Discrepancies were brought to a third author (SR) and discussed until a consensus was reached. Kappa and Cohen's scores were calculated to establish concordance in the title, abstract, and full article selection. In a process known as reference snowballing [16], the authors manually searched the final 108 eligible article references and included five additional studies that met the inclusion criteria.

Data Extraction and Quality Assessment

Data extraction included patient demographics such as age and sex, in addition to bone type, length of follow-up, operative technique, specific NSAID used, level of evidence, and the primary outcomes of interest. Study quality was independently assessed to identify any potential bias. The Newcastle-Ottawa Scale (NOS) was employed for non-randomized studies, while the Cochrane Collaboration Risk of Bias 2 (RoB 2) scale was used for randomized studies [17,18]. Scores were converted to values of 0, 1, and 2, respectively, to facilitate data sorting. Studies with a NOS below 5 on the 9-point scale or a Cochrane Collaboration RoB 2 score of 6 or higher were excluded from the analysis. We also excluded any study that showed zero events in either the NSAID-exposed or non-exposed group [19].

Statistical Analysis

This meta-analysis adhered to the QUOROM [20] and PRISMA guidelines [21]. Data analysis was performed using R with the metafor package (RStudio, Boston, MA) [22]. A maximum likelihood random-effects model to minimize variance was implemented. Pooled effect estimates were expressed as odds ratios (OR) with 95% confidence intervals (CIs) using the maximum likelihood random-effects model. The OR for each study was calculated using the number of abnormal bone healing events and nonevents with versus without NSAID use. All analyses had a significance level (α) set at 0.05.

Results

**Figure 2:**
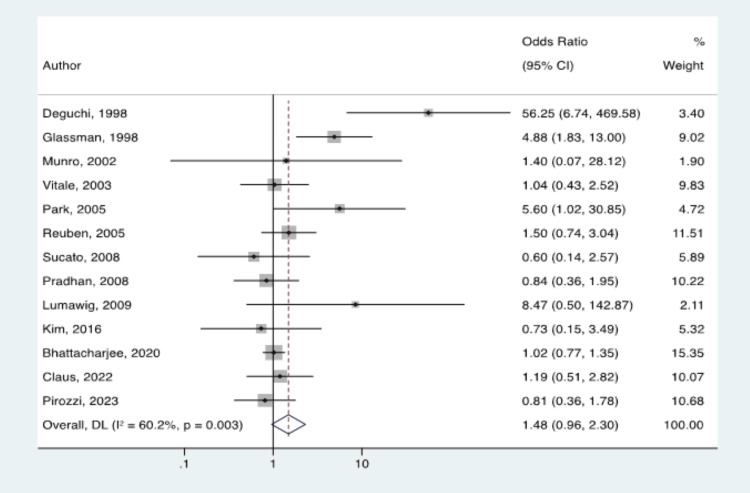
Forest plot of odds ratios for adverse bone healing with versus without NSAIDs use after arthrodesis for included studies Deguchi et al. [32], Glassman et al. [31], Munro et al. [27], Vitale et al. [35], Park et al. [33], Reuben et al. [26], Sucato et al. [34], Pradhan et al. [25], Lumawig et al. [30], Kim et al. [24], Bhattacharjee et al. [29], Claus et al. [28], Pirozzi et al. [23].

After removing duplicates, we screened a total of 3,050 articles. One hundred and eight full-text articles were assessed for eligibility in the meta-analysis, of which eight met inclusion criteria [23-30] (Table 1). The chance-adjusted kappa scores between the two authors (ER and JTS) for the title screen, abstract screen, and full-text screen were 0.71, 0.78, and 0.84, respectively. Through a method known as snowballing [16], a manual search of references from the 108 articles identified five additional papers that met inclusion criteria and were also included in the final analysis [31-35] (Table 1). Notably, all the included studies met the minimum bias requirements. Included were 12 spines and one foot and ankle study. Ten were retrospective case-control studies, and three were randomized control trials (RCTs). These 13 studies were included in the final analysis and underwent final data extraction. The average patient age was 42 (SD 20), and the minimum follow-up in months was 16 (SD 7.6). The total cohort included 9,929 arthrodesis procedures, of which 3,551 were exposed to NSAIDs and 6,378 were not. There were 522 reported cases of abnormal bone healing, of which 251 (7.07%) were exposed to NSAIDs and 271 (4.25%) were not. The resulting statistical analysis of the study pool gave an OR of 1.485 with a 95% CI of 0.957 to 2.304, indicating a non-significant relationship between NSAID exposure and abnormal bone healing (Figure 2). The individual ORs in the studies reviewed ranged from 0.604 to 56.25.

**Table 1:** Demographic and study characteristics of studies included in the meta-analysis RCT: randomized controlled trial

Author	Level of evidence	Study type	Total sample size	Mean age	Operative technique	# Control normal healing	# NSAID normal healing	# Control delayed or non-union	# NSAID delayed or non-union
Deguchi et al. [32]	3	Retrospective cohort	83	38	Spinal fusion	45	12	1	15
Glassman et al. [31]	4	Retrospective cohort	288	43.8	Spinal fusion	116	138	5	29
Munro et al. [27]	1	RCT	35	14	Spinal fusion	7	5	1	1
Vitale et al. [35]	3	Retrospective cohort	208	13.4	Spinal fusion	128	52	19	8
Park et al. [33]	4	Retrospective cohort	88	53	Spinal fusion	56	25	2	5
Reuben et al. [26]	2	Retrospective cohort	434	46.1	Spinal fusion	119	267	11	37
Sucato et al. [34]	3	Retrospective cohort	319	14.3	Spinal fusion	156	155	5	3
Pradhan et al. [25]	3	Retrospective cohort	405	56.2	Spinal fusion	166	216	11	12
Lumawig et al. [30]	3	Retrospective cohort	273	-	Spinal fusion	19	209	0	45
Kim et al. [24]	1	RCT	80	67.1	Spinal fusion	36	37	4	3
Bhattacharjee et al. [29]	3	Retrospective cohort	7315	-	Spinal fusion	5060	2012	173	70
Claus et al. [28]	1	RCT	246	61.2	Spinal fusion	117	107	11	12
Pirozzi et al. [23]	3	Retrospective cohort	155	55.6	Foot/ankle fusion	82	40	28	11

**Figure 3:**
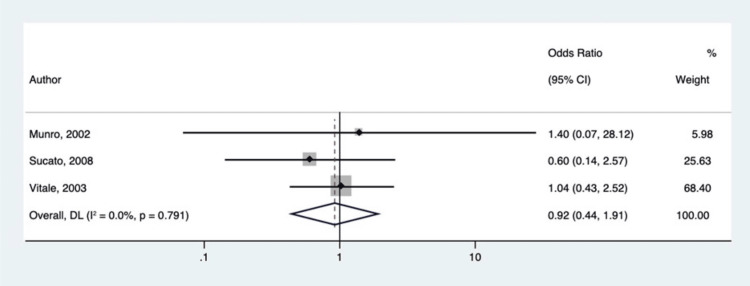
Forest plot of odds ratios for adverse bone healing with versus without NSAIDs use after arthrodesis for only pediatric studies Munro et al. [27], Sucato et al. [34], Vitale et al. [35].

Subgroup analysis of pediatric and adult studies demonstrated significant differences between the two populations. Three studies (n = 562) were included for pediatric subgroup analysis. Of the 224 patients exposed to NSAIDs, only 12 experienced abnormal bone healing (5.35%). The remaining 291 without exposure to NSAIDs had 12 abnormal bone healing events (4.12%). Statistical analysis for our pediatric studies showed that NSAIDs do not negatively affect the bone healing process in pediatric patients (p = 0.821) and yielded an OR of 0.919 and a 95% CI of 0.441 to 1.913 (Figure 3).

**Figure 4:**
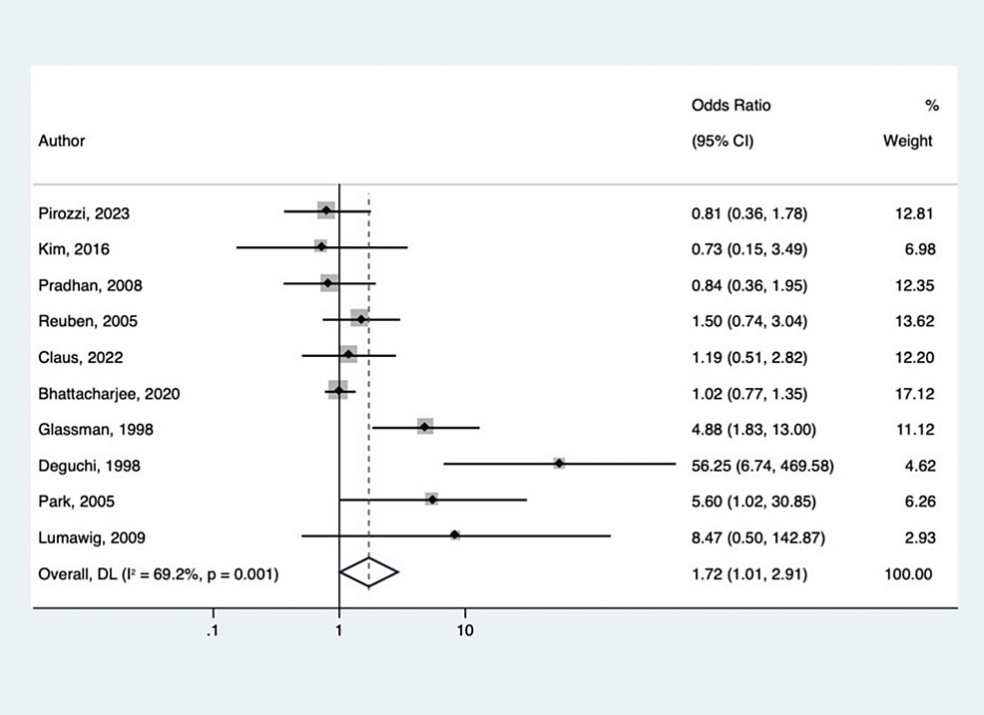
Forest plot of odds ratios for adverse bone healing with versus without NSAIDs use after arthrodesis for only adult studies Pirozzi et al. [23], Kim et al. [24], Pradhan et al. [25], Reuben et al. [26], Claus et al. [28], Bhattacharjee et al. [29], Glassman et al. [31], Deguchi et al. [32], Park et al. [33], Lumawig et al. [30].

The adult cohort, on the other hand, did reveal significant findings with regard to fusion rates with versus without NSAID use. The 10 studies combined for a sample size of 9,367. In total, 3,302 patients were exposed to NSAIDs, and 239 of them experienced abnormal bone healing (7.24%). Of the 6,062 who were not exposed to NSAIDs, 246 still experienced an adverse bone healing event (4.06%). The statistical analysis showed that NSAIDs significantly increased the rate of abnormal bony healing in adults (p = 0.045) with an OR of 1.717 and a 95% CI of 1.012 to 2.914 (Figure 4).

Discussion

After an extensive review of the current literature, this meta-analysis assesses the impact of NSAID exposure on union rates following arthrodesis procedures, differentiating between pediatric and adult patients. Contrary to the study's hypothesis, the findings indicate that postoperative NSAID use does significantly increase the risk of nonunion in adult arthrodesis cases, while no notable association is observed in pediatrics. The diverse range of odds ratios across the included studies highlights the heterogeneity in NSAID administration, variations in bone healing among patients, and the influence of confounding variables. This underscores the need for a standardized approach to NSAID administration tailored to each patient's unique characteristics.

In 2010, Dodwell et al. conducted one of the initial meta-analyses, comprehensively examining the association between NSAID exposure postoperatively and the risk of nonunion. In their preliminary analysis of 11 cohort and case-control studies, the pooled OR showed a significant relationship between nonunion and NSAID exposure (OR, 3.0; 95% CI, 1.6-5.6) [36]. However, this finding included arthrodesis and fracture studies in one study. Sub-analysis of their findings into high- and low-quality studies yielded seven spine arthrodesis studies, which, when analyzed, showed no statistically significant association between NSAID exposure and non-union (OR, 2.2; 95% CI: 0.8-6.3). All seven of their spine arthrodesis studies were included in this meta-analysis, which, when pooled, similarly showed no statistically significant increase in non-union risk. However, the 2010 meta-analysis does not analyze non-union risk based on age, overlooking the well-understood difference in bone healing between adult and pediatric patients [37]. This study’s emphasis on age sub-analysis provides evidence that considering age as a risk factor for non-union following joint arthrodesis is crucial.

Similar to this sub-analysis of adult and pediatric non-union findings, a 2018 meta-analysis performed by Wheatley et al. demonstrated that inflammatory inhibition by NSAIDs has the potential to yield unfavorable results in adult patients. Their total pooled adult cohort showed a significantly increased risk of abnormal bone healing with NSAID use after arthrodesis (OR, 4.9; 95% CI, 1.45-16.58) [13]. However, they included five arthrodesis studies in their analysis of adult cohorts, while this study's updated meta-analysis incorporates five additional studies. The subgroup analysis from this meta strengthens the claims made in the 2018 meta-analysis because the adult sub-analysis showed a significantly increased risk for non-union following arthrodesis procedures (OR, 1.72; 95% CI, 1.01-2.91). Notably, all five arthrodesis studies from the research by Wheatley et al. are included in this updated meta-analysis, which similarly shows a significant relationship between exposure to NSAIDs and bone healing following arthrodesis procedures. Furthermore, this analysis addresses a limitation of the previous meta-analysis, which noted the absence of RCTs. In contrast, this study includes three studies with level 1 evidence, contributing to the overall robustness of the evidence presented and providing valuable insights to guide orthopedic surgeons in understanding the safety profile of post-operative NSAID use following arthrodesis procedures.

This study’s findings in the pediatric subgroup analysis correspond to findings made by another meta-analysis reviewing pediatric spinal arthrodesis healing when exposed to NSAIDs that showed no correlation between NSAID use and an increased risk of abnormal bony healing [38]. Additionally, the findings by Wheatley et al. in the 2018 subgroup analysis of pediatric patients found no correlation between NSAID use and abnormal bone healing (OR, 0.58; 95% CI, 0.27-1.21) [13]. Their cohort of pediatric patients included two fracture studies and two arthrodesis studies, both of which were incorporated into the present meta-analysis. Consistent with the results here, their analysis similarly indicates no adverse impact of NSAIDs on bone healing in pediatric patients following arthrodesis procedures. With the inclusion of one additional pediatric arthrodesis study in this meta-analysis, the collective evidence from all three studies supports the use of NSAIDs in pediatric patients. Current literature attributes this low-risk profile in large part to the robust inflammatory response found in skeletally immature healing responses [39]. These findings should instill confidence in surgeons when exploring alternatives to opioids in pediatric arthrodesis pain management.

The strength of this study lies in perceiving the effect on humans alone for directly applicable results. One of the more recently published studies looking at the effect of NSAIDs on spinal arthrodesis combined animal studies and human studies into one cohort [8], confusing what could be an abnormal healing event by animals vs. humans, given their different healing capacities [40]. Additionally, the present meta-analysis is comprised of high-quality studies, including three RCTs, contributing to the strength of this study. This meta-analysis also had a large sample patient population, despite the limited number of included studies. Limitations of the present meta-analysis include a lack of subgroup analysis of specific types of NSAIDs and dosages of medication. The ability to identify differences between low and high doses of NSAIDs could further elucidate the overall safety of administering NSAIDs post-operatively. Furthermore, it is important to interpret the findings of this study with the awareness that variables such as the type of NSAID administered, dosage, and administration methods were not controlled for. The absence of control for these factors raises the possibility of unavoidable confounding variables, which could potentially influence the association between NSAIDs and bone healing following arthrodesis procedures. However, the use of the random-effects model should potentiate some differences. The study did not control for the types of bones or the location of arthrodesis. The varying locations, types of bones, and levels fused in spinal arthrodesis may exhibit different inflammatory properties or responses. Another limitation was the lack of consideration for smoking status within all studies, given that smoking is one of the greatest risk factors for poor bone healing following fractures and arthrodesis [41].

In addition to the above-noted limitations of this meta-analysis, limitations stemming from potential bias must be considered. Many of the reviewed articles showed potential bias despite the use of quality thresholds. Patients undergoing spinal arthrodesis across multiple levels may encounter elevated pain scores, potentially leading to increased NSAID exposure. This heightened exposure could pose a risk for non-unions, suggesting the presence of selection bias. Given the retrospective nature of many included studies, there is a possibility of recall bias. Patients experiencing unfavorable outcomes, such as non-unions, may be more likely to recall and emphasize exposures of interest when asked about specific details. Furthermore, despite the systematic approach to the applied literature search in this meta, there is a non-zero possibility of overlooking articles that met the inclusion requirements for this study. The exclusion of non-English articles introduces the potential for missed, relevant studies to be included.

## Conclusions

This updated meta-analysis supports the notion that NSAIDs may significantly impact union rates, specifically in the adult population, following arthrodesis procedures. Nevertheless, the findings in this meta-analysis do support the safe use of NSAIDs as part of the postoperative pain regimen in pediatric patients who have had an arthrodesis procedure. Prior to this study, most recent meta-analyses have advised against NSAID use for post-operative pain control after arthrodesis procedures due to concerns about the potential risk of delayed union or nonunion. However, individual study results vary in their recommendations for NSAID utilization. Owing to the heterogeneous types, dosages, and administration patterns of NSAIDs in the setting of pain management and the differences among arthrodesis locations or bones fused, a definitive answer regarding the NSAID effect on non-union following arthrodesis cannot be provided. Without control for various bone types or specific procedures, truly patient-specific pain management regimens using NSAIDs cannot be achieved. Future studies, particularly RCTs that employ a standardized pain management regimen, observe a sub-analysis of dose-dependent responses, specify the NSAIDs used, and consider the timing of administration, would provide the most robust evidence to better elucidate ideal postoperative pain management strategies after arthrodesis procedures.

## Appendices

Literature search

**Table 2:** Search strategy for literature search Publication date from January 1, 1990 to January 6, 2023.

Database	Bones	Subjects	Injury	Exposure	Outcome	Time Factors
Ovid	Bony OR Bone OR Ankle Bone OR Arm Bone OR Foot Bone OR Hand Bone OR Wrist Bone OR Leg Bone OR Pelvic Bone OR Hip Bone OR Facial Bone OR Femoral OR Femur OR Fibular OR Fibula OR Humeral OR Humerus OR Radial OR Radius OR Tibial OR Tibia OR Ulnar OR Ulna OR Spinal OR Spine OR Vertebral OR Vertebra OR Sacral OR Sacrum OR Costal OR Rib OR Clavicular OR Clavicle OR Carpal OR Metacarpal OR Phalange OR Tarsal OR Metatarsal	Human	Fusion OR Fusion Surgery OR Arthrodesis OR Pseudoarthrosis	Anti-Inflammatory Agents, Non-Steroidal OR indomethacin OR Ketorolac OR Ibuprofen OR Naproxen OR Diclofenac OR Celecoxib OR Mefenamic Acid OR Etoricoxib OR Nonsteroid anti-inflammatory agent OR Nonsteroid antiinflammatory agent OR NSAID	Union OR Nonunion OR Non-union OR Non-bone union OR Healing OR Malunited OR Ununited OR United OR Adverse Bone Healing OR Drug effects OR Recovery of Function	(day OR days) OR (week OR weeks) OR (month OR months) OR (year OR years) OR Time OR Time factors
Embase	Bony OR Bone OR Bones OR (Arm Bones) OR (Foot Bones) OR (Hand Bones) OR (Wrist Bones) OR (Leg Bones) OR (Pelvic Bones) OR (Hip Bones) OR (Facial Bone) OR Femoral OR Femur OR Fibular OR Fibula OR Humeral OR Humerus OR Radial OR Radius OR Tibial OR Tibia OR Ulnar OR Ulnar OR Spinal OR Spine OR Vertebral OR Vertebra OR Sacral OR Sacrum OR Costal OR Rib OR Clavicular OR Clavicle OR Carpal OR Metacarpal OR Phalange OR Tarsal OR Metatarsal	Human	Fusion OR (Fusion Surgery) OR Arthrodesis OR Pseudoarthrosis	(Anti-Inflammatory Agents, Non-Steroidal) OR Indomethacin OR Ketorolac OR Ibuprofen OR Naproxen OR Diclofenac OR Celecoxib OR Mefenamic Acid OR Etoricoxib OR (Nonsteroid anti-inflammatory agent) OR (Nonsteroid antiinflammatory agent) OR NSAID	Union OR Nonunion OR Non-union OR (Non-bone union) OR Healing OR Malunited OR Ununited OR United OR (Adverse Bone Healing) OR Drug effects OR (Recovery of Function)	(day OR days) OR (week OR weeks) OR (month OR months) OR (year OR years) OR Time OR (Time factors)
Web of Science	Bony OR Bone OR Bones OR Femoral OR Femur OR Fibular OR Fibula OR Humeral OR Humerus OR Radial OR Radius OR Tibial OR Tibia OR Ulnar OR Ulnar OR Spinal OR Spine OR Vertebral OR Vertebra OR Sacral OR Sacrum OR Costal OR Rib OR Clavicular OR Clavicle OR Carpal OR Metacarpal OR Phalange OR Tarsal OR Metatarsal	Human	Fusion OR (Fusion Surgery) OR Arthrodesis OR Pseudoarthrosis	(Anti-inflammatory Agents, Non-Steroidal) OR Indomethacin OR Ketorolac OR Ibuprofen OR Naproxen OR Diclofenac OR Celecoxib OR Mefenamic Acid OR Etoricoxib OR (Nonsteroid anti-inflammatory agent) OR (Nonsteroid antiinflammatory agent) OR NSAID	Union OR Nonunion OR Non-union OR (Non-bone union) OR Healing OR Malunited OR Ununited OR United OR (Adverse Bone Healing) OR Drug effects OR (Recovery of Function)	(day OR days) OR (week OR weeks) OR (month OR months) OR (year OR years) OR Time OR (Time factors)
Cochrane Library	Bony OR Bone OR Bones OR Femoral OR Femur OR Fibular OR Fibula OR Humeral OR Humerus OR Radial OR Radius OR Tibial OR Tibia OR Ulnar OR Ulnar OR Spinal OR Spine OR Vertebral OR Vertebra OR Sacral OR Sacrum OR Costal OR Rib OR Clavicular OR Clavicle OR Carpal OR Metacarpal OR Phalange OR Tarsal OR Metatarsal	Human	Fracture OR Broken OR Fusion OR Fusion NEXT Surgery OR Arthrodesis OR Pseudoarthrosis	Mesh (Anti-Inflammatory Agents, Non-Steroidal) OR Indomethacin OR Ketorolac OR Ibuprofen OR Naproxen OR Diclofenac OR Celecoxib OR Mefenamic NEXT Acid OR Etoricoxib OR (Nonsteroid NEXT anti-inflammatory NEXT agent OR Nonsteroid NEXT antiinflammatory NEXT agent OR NSAID)	Union OR Nonunion OR Non-union OR Non-bone NEXT union OR Healing OR Malunited OR Ununited OR United OR Adverse NEXT Bone NEXT Healing OR Drug NEXT effects OR Recovery NEXT of NEXT Function	Day OR days OR week OR weeks OR month OR months OR year OR years OR Time OR Time NEXT factors
